# Association of retinal microvascular abnormalities and neuromyelitis optica spectrum disorders with optical coherence tomography angiography

**DOI:** 10.3389/fnins.2023.1194661

**Published:** 2023-06-09

**Authors:** Jiaqi Guo, Dan Zhang, Yan Gong, Jiang Liu, Jiong Zhang, Yitian Zhao

**Affiliations:** ^1^Cixi Institute of Biomedical Engineering, Ningbo Institute of Materials Technology and Engineering, Chinese Academy of Sciences, Ningbo, China; ^2^University of Chinese Academy of Sciences, Beijing, China; ^3^School of Cyber Science and Engineering, Ningbo University of Technology, Ningbo, China; ^4^The Affiliated Ningbo Eye Hospital of Wenzhou Medical University, Ningbo, China; ^5^Department of Computer Science and Engineering, Southern University of Science and Technology, Shenzhen, China

**Keywords:** OCTA, retinal microvascular, neuromyelitis optica spectrum disorders, deep learning, segmentation

## Abstract

**Introduction:**

Neuromyelitis optica spectrum disorders (NMOSD) are autoimmune central nervous system diseases characterized by the immune system's abnormal attack on glial cells and neurons. Optic neuritis (ON) is one of the indicators of NMOSD, often starting unilaterally and potentially affecting both eyes later in the disease progression, leading to visual impairment. Optical coherence tomography angiography (OCTA) has the potential to aid in the early diagnosis of NMOSD by examining ophthalmic imaging and may offer a window for disease prevention.

**Methods:**

In this study, we collected OCTA images from 22 NMOSD patients (44 images) and 25 healthy individuals (50 images) to investigate retinal microvascular changes in NMOSD. We employed effective retinal microvascular segmentation and foveal avascular zone (FAZ) segmentation techniques to extract key OCTA structures for biomarker analysis. A total of 12 microvascular features were extracted using specifically designed methods based on the segmentation results. The OCTA images of NMOSD patients were classified into two groups: optic neuritis (ON) and non-optic neuritis (non-ON). Each group was compared separately with a healthy control (HC) group.

**Results:**

Statistical analysis revealed that the non-ON group displayed shape changes in the deep layer of the retina, specifically in the FAZ. However, there were no significant microvascular differences between the non-ON group and the HC group. In contrast, the ON group exhibited microvascular degeneration in both superficial and deep retinal layers. Sub-regional analysis revealed that pathological variations predominantly occurred on the side affected by ON, particularly within the internal ring near the FAZ.

**Discussion:**

The findings of this study highlight the potential of OCTA in evaluating retinal microvascular changes associated with NMOSD. The shape alterations observed in the FAZ of the non-ON group suggest localized vascular abnormalities. In the ON group, microvascular degeneration in both superficial and deep retinal layers indicates more extensive vascular damage. Sub-regional analysis further emphasizes the impact of optic neuritis on pathological variations, particularly near the FAZ's internal ring.

**Conclusion:**

This study provides insights into the retinal microvascular changes associated with NMOSD using OCTA imaging. The identified biomarkers and observed alterations may contribute to the early diagnosis and monitoring of NMOSD, potentially offering a time window for intervention and prevention of disease progression.

## 1. Introduction

Neuromyelitis optica spectrum disorders (NMOSD), also known as Devic's disease or Devic's syndrome, is a rare autoimmune central nervous system (CNS) disease that predominantly affects the optic nerves and the spinal cord (Wingerchuk et al., 2007; De Seze et al., 2008). NMOSD is a demyelinating disease that can cause irreversible damage to the nervous system. The onset of NMOSD often begins in the unilateral eye or spinal cord and has a high relapse rate as well as disability rate. Patients will experience symptoms such as vision loss and paralysis. While NMOSD was previously diagnosed as a variant of multiple sclerosis (MS), it is now known to have distinct immunological features. In many cases, NMOSD is characterized by the antibody of the water channel aquaporin-4 (AQP4) of the astrocytic in CNS (Fernandes et al., 2013). The current diagnostic method for NMOSD combines serum testing information and NMR information in practice. However, a correct diagnosis remains challenging as AQP4 antibodies cannot be detected in a minority of NMOSD patients. Thus, an early and accurate diagnostic approach of NMOSD is crucial (Fernandes et al., 2013).

Since NMO-related optic neuritis directly affects visual ability, ophthalmological examinations may be helpful to assist clinicians in the diagnosis of NMOSD (Lang et al., 2023; Xie et al., 2023). Optical coherence tomography (OCT) is a non-invasive technique that provides a convenient means for the clinical analysis of retinal structures (Merle et al., 2008). Over the last years, several studies have suggested that retinal nerve fiber layer (RNFL) and macular thickness analysis using OCT are valuable for the detection and analysis of axonal loss and treatment effects monitoring in MS and NMOSD (Nakamura et al., 2010; Lange et al., 2013; Lee et al., 2020). Moreover, previous OCT studies have shown that optic neuritis (ON) in NMOSD causes more severe neuronal damage and an obvious decrease of the RNFL than in MS (Monteiro et al., 2012; Schneider et al., 2013; Papadopoulou et al., 2021), which are consistent with the clinical findings that the visual loss in NMOSD is usually more severe than in MS.

In addition to the analysis of different retinal layers, the geometric and topological features of retinal microvasculature can also provide valuable information for the early diagnosis of NMOSD (Ghassemi et al., 2017). In recent years, Optical coherence tomography angiography (OCTA) has been emerging as a non-invasive technique for imaging the retinal microvasculature (Kleerekooper et al., 2020). The OCTA imaging is generated by analyzing the signal differences between repeated OCT scans in a short instant, thus can detect moving red blood cells to accurately depict the depth-resolved microvascular details. In clinical practice, OCTA can be explored in various ways to advance the understanding of neuroinflammatory diseases such as MS (Yilmaz et al., 2020). Previous OCTA studies for neuroinflammatory disease have found that its microvascular measurements are usually highly correlated with the measurements of retinal layer thickness from OCT (Wang et al., 2014; Ann et al., 2018) In addition to that, several studies (Rogaczewska et al., 2021a,b,c) have shown that the OCTA microvascular metrics are more effective in distinguishing NMOSD patients from MS than using OCT-based metrics such as the RNFL thickness.

The objective of our study is to establish an effective approach for extracting and analyzing important OCTA microvascular measures from all retinal layers to find their associations with NMOSD. To this end, we first build up a processing pipeline for achieving accurate microvascular and FAZ segmentation. Afterwards, we define 12 important microvascular metrics to quantitatively measure the morphological changes. By performing dedicated statistical comparisons with healthy controls (HC), we can better correlate the most significant vessel biomarkers with the disease progression and explore their potential for early diagnosis. Besides that, we also utilize the Early Treatment Diabetic Retinopathy Study (ETDRS) macular grid to perform a sub-region analysis to investigate the microvascular changes in terms of different regions, as it has been shown in multiple studies (Age-Related Eye Disease Study Research Group, 2001; Forshaw et al., 2021) to allow researchers to further understand the impact of disease on different subregions of the retina.

## 2. Method

The clinical protocol of our study was approved by the ethics committee of the Cixi Institute of Biomedical Engineering, Chinese Academy of Sciences, and adhered to the principles of the Declaration of Helsinki. Participants enrolled in our study have provided informed written consent.

### 2.1. NMOSD participants and healthy controls

Our study enrolled a total of 22 participants diagnosed with neuromyelitis optica spectrum disorders (NMOSD) and 25 healthy control (HC) participants. The diagnosis of NMOSD was confirmed by the Affiliated Ningbo Eye Hospital of Wenzhou Medical University, Ningbo, China. The dataset consisted of 44 and 50 eyes from the NMOSD and HC participants, respectively. The diagnosis of all participants was based on clinical examination by experts following the diagnostic criteria for NMOSD, including immunology and MRI examinations. NMOSD has an even stronger female predilection than multiple sclerosis (Wingerchuk, 2009; Pandit et al., 2015), all participants in this study are female. NMOSD and HC participants also have a similar stage of educational background and age, as shown in Table 1. Participants with pathological conditions such as hypertension, uncontrolled hypertension, neurological disorders, and current or previous substance abuse were excluded from the study. Table 1 shows that there was no significant difference(*P*≥0.050) in age and body mass index (BMI) between the NMOSD and HC groups. However, the data shows that the best corrected visual acuity (BCVA) of the NMOSD patients group was significantly lower (*P* < 0.050) than that of the HC group, indicating that visual impairment is one of the symptoms of NMOSD.

**Table 1:** Results of *t*-test for demographic information.

**Variable**	**HC**	**NMOSD**	***P***
Age	47.36 (13.21)	47.68 (12.48)	0.904
Best corrected visual acuity	1.15 (0.18)	0.66 (0.47)	<**0.001**
Body mass index	22.80 (2.51)	22.34 (2.38)	0.385

The data with *P* value less than 0.05 as bold value, which generally means that the parameters they represent are significant.

### 2.2. OCTA image acquisition

All participants were imaged using the RTVue XR Avanti SD-OCT system (Optovue, USA) with a speed of 70,000 A-scans per second. The field of view (FOVs) was set as 3 × 3 mm on the fovea. The en-face images of the superficial vascular complex (SVC), deep vascular complex (DVC), and intermediate vascular complex (IVC) with a resolution of 304 × 304 pixels were exported for analysis. Images with low signal strength intensity (SSI), artifacts, and other obvious problems were excluded from our data analysis.

**Figure 1:**
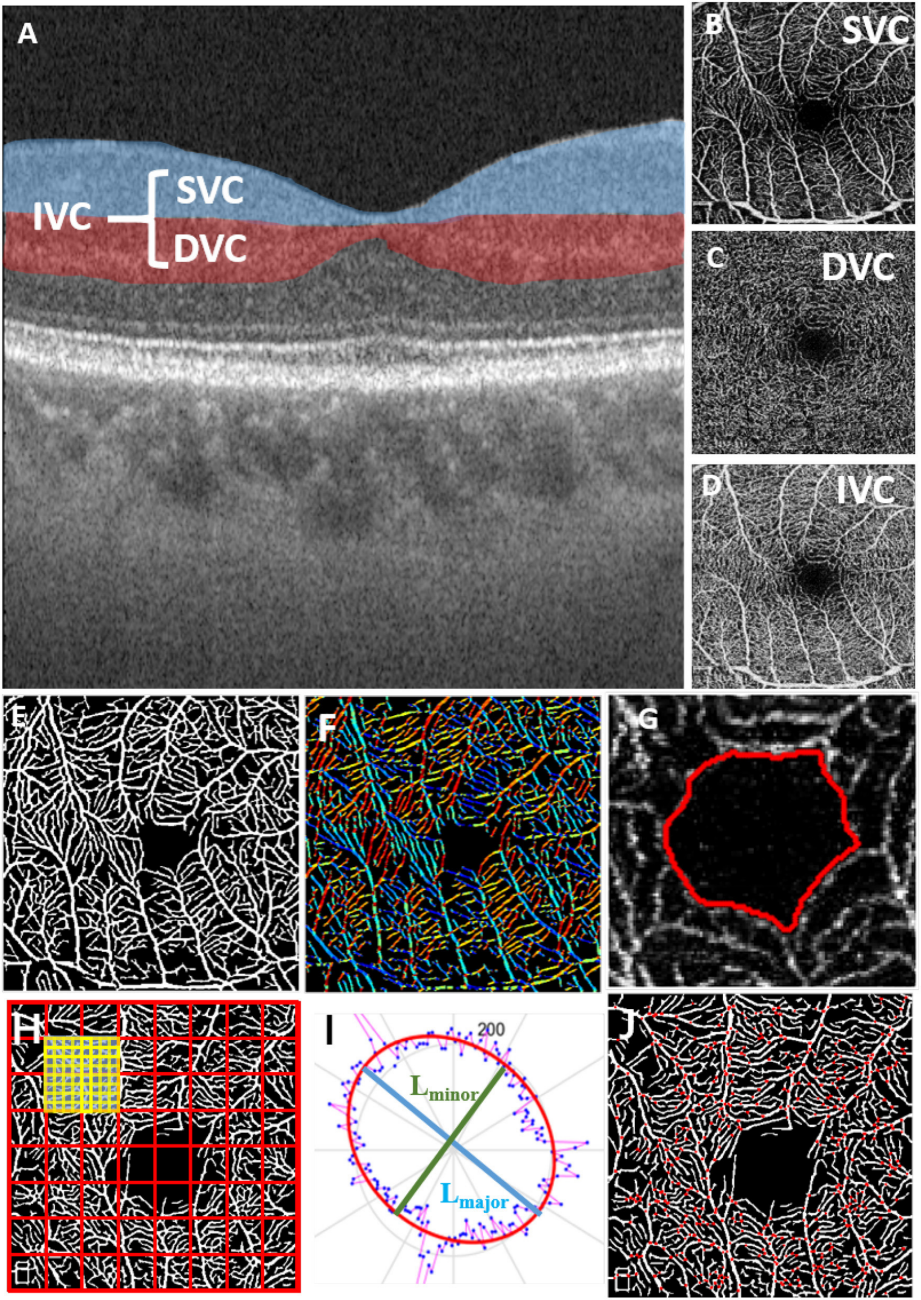
Microvascular and FAZ-related parameters for quantitative measurements in different OCT layers; **(A)** OCT layers; **(B)** superficial vascular complex (SVC); **(C)** deep vascular complex (DVC); **(D)** intermediate vascular complex (IVC); **(E)** result of retinal microvascular segmentation; **(F)** vessel orientations indicated by different colors; **(G)** FAZ segmentation result; **(H)** fractal dimension calculation; **(I)** ellipse fitted by the vessel orientation; **(J)** vessel bifurcation point extraction.

The superficial vascular complex (SVC), deep vascular complex (DVC), and intermediate vascular complex (IVC) are different layers of the retinal vasculature that can be visualized using optical coherence tomography angiography (OCTA). The SVC is located in the innermost layer of the retina and is composed of capillaries that supply blood to the nerve fiber layer. The DVC is located in the outermost layer of the retina and is composed of capillaries that supply blood to the photoreceptor layer. The IVC is located between the SVC and DVC and is composed of capillaries that supply blood to the inner nuclear layer (Figures 1A–D).

### 2.3. Microvascular and foveal avascular zone (FAZ) segmentation

To achieve effective biomarker analysis using OCTA images, we need to first precisely extract the key structures such as retinal microvasculature and FAZ, which is a region within the fovea centralis at the center of the retina of the eye that is devoid of retinal blood vessels. Since traditional methods show stronger robustness in vessel segmentation of OCTA images, we followed and improved the previous work of our laboratory, the infinite active contour model (Zhao et al., 2015) to segment the retinal microvasculature and FAZ from OCTA images. This model has been applied to three publicly available retinal datasets and performed well. In this method, an infinite perimeter regularizer allows for better detection of small microvasculature. Moreover, we use different types of region information, such as the combination of intensity information and local phase based enhancement map. The local phase based enhancement map is used for its superiority in preserving vessel edges, while the given image intensity information will guarantee a correct feature's segmentation. In Figure 1, we show the result of retinal microvascular segmentation (Figure 1E) and FAZ segmentation (Figure 1G).

### 2.4. Definition of quantitative parameters

We aim to measure 12 important parameters that describe the morphological changes of both microvasculature and FAZ, as illustrated in Figure 1.

- FAZ area (FA): given the relative size of the FAZ area (the area enclosed by the red line in Figure 1G);
- FAZ circularity (FC): represents the degree of roundness of the FAZ. The larger FC, the more circular of the shape, and a value of 1 denotes a perfect circle (the length of red line in Figure 1G);
- FAZ axial ratio (FAR): calculates the ratio between the major and minor axes of the fitted ellipse from the FAZ boundary. A higher FAR indicates an elongated FAZ with greater eccentricity (the ratio of the major diameter to the minor diameter of the ellipse fitted by the red line in Figure 1G);
- FAZ roundness (FR): is similar to FC, but is less sensitive to irregular borders along the perimeter of FAZ;
- FAZ solidity (FS): describes to which degree the FAZ is convex or concave, and is defined as the ratio between the FA and the convex area covering the FAZ. The further the solidity deviates from 1, the greater the extent of concavity in the structure.
- Direction area: gives the area of ellipse which is fitted according to the direction of all pixels of the segmented microvasculature (Figure 1F);
- Direction ratio: gives the axial ratio of the ellipse which is fitted according to the direction of all pixels in the segmented microvasculature (Figure 1I);
- Tortuosity: given the measure of the tortuous level of the microvasculature;
- VAD: the total length in millimeters of the perfused retinal microvasculature per unit area in square millimeters in the annular region of the analyzed area (the segmentation results of retinal microvasculature are shown in Figure 1E);
- VLD: represents the ratio between the total number of pixels of microvascular centerline and the area of analyzed region;
- Fractal Dimension (FD): A well-known measure of the geometric complexity of microvasculature, the calculation method comes from Fraclab, a third-party toolbox of MATLAB, as shown in (Figure 1H);
- Bifurcation number (B-num): counting the number of vessel bifurcation points in the image (Figure 1J).

### 2.5. Definition of sub-regions

**Figure 2:**
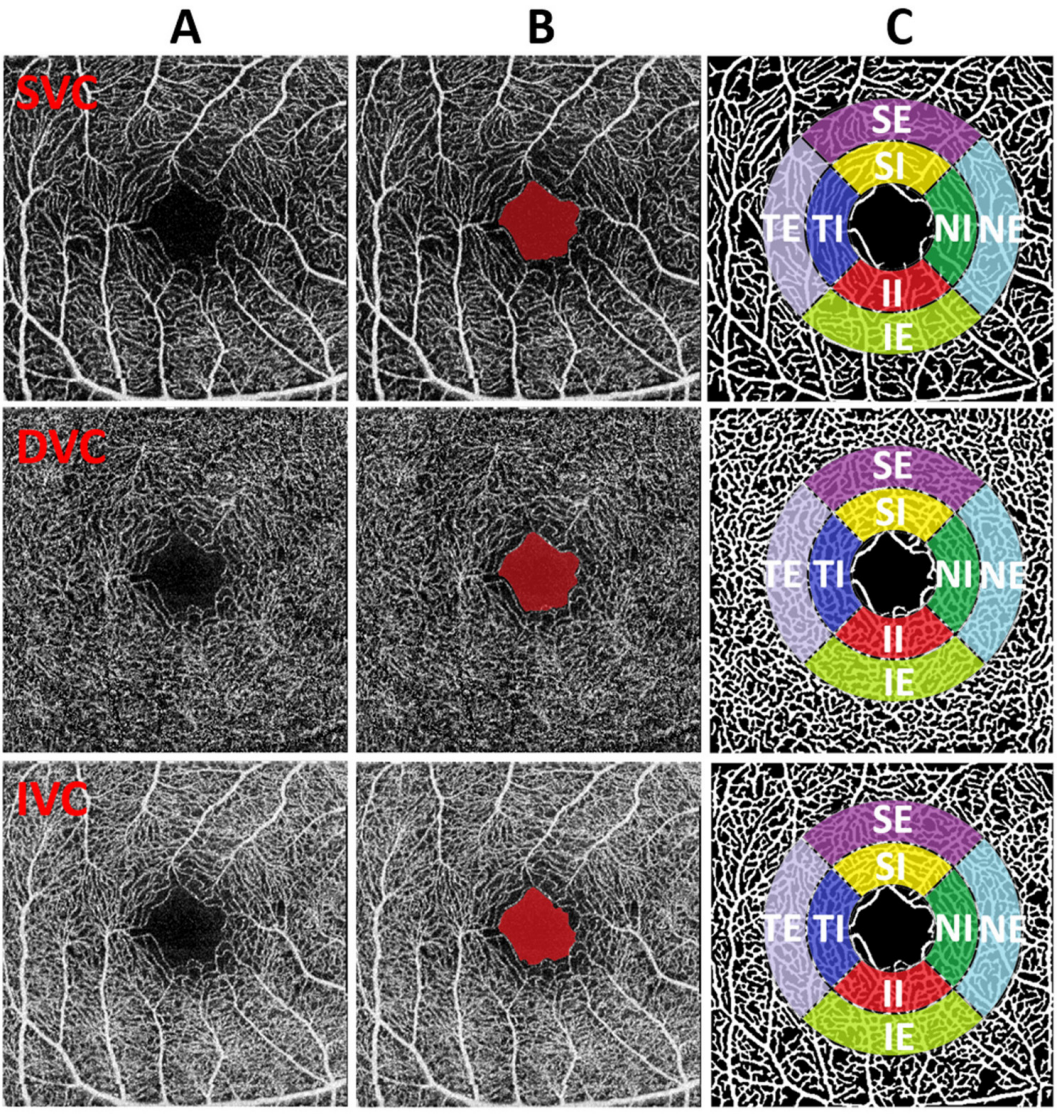
**(A–C)** Illustration of FAZ and sub-region analysis. From top to the bottom, the image of different plexus (SVC, DVC, and IVC) are shown respectively. From left to right: original images, FAZ results (red mark), original image with the overlay of sub-regions on vascular segmentation results.

We will utilize the Early Treatment Diabetic Retinopathy Study (ETDRS) to perform specific sub-regional analysis of VAD, VLD, Fractal Dimension (FD), Tortuosity, and B-num. The purpose is to explore the influence of NMOSD patients' ON on the morphological changes of retinal microvasculature. The definitions of the ETDRS for the eight macular retinal sub-regions are shown in Figure 2. The FAZ area is defined as the partition center with a diameter of *R* = 0.75 mm. Then an internal ring and an external ring with respective diameters of 2 × *R* and 3 × *R* are defined as candidate regions for analysis. Furthermore, these regions are divided into 4 quadrants and are thus recognized as 8 sectors named superior-internal (SI), temporal-internal (TI), inferior-internal (II), nasal-internal (NI), superior-external (SE), temporal-external (TE), inferior-external (IE), and nasal-external (NE) for sub-regional analysis.

## 3. Results

### 3.1. Statistical comparisons between the NMOSD and HC groups

We first compute all 12 OCTA microvascular and FAZ metrics with their segmentation results. These metrics are respectively calculated on the SVC, DVC, and IVC layers. Then, we perform statistical comparisons between the NMOSD group and HC group for each metric. The results are shown in Table 2. In the SVC of the NMOSD group, there are significant increases in terms of FA and tortuosity, and decreases in terms of the direction area, VAD, VLD, FD, and B-num, with a statistical significance of *P* < 0.050 compared with the HC group. The FC, FAR, FR, FS, and direction ratio of the two groups show no significant statistical difference. In the DVC layer, the NMOSD group also presents increases in FA and decreases in direction area, VAD, VLD, FD, and B-num. However, there is no significant difference in the statistical distributions of tortuosity, FC, FAR, FR, FS, and Direction-ratio. In the IVC layer, there is no noticeable statistical difference in all metrics between the NMOSD and NC groups.

**Table 2:** Comparisons of OCTA parameters between HC with NMOSD participants.

**Variable**	**SVC**			**DVC**			**IVC**		
	**NMOSD**	**HC**	**P**	**NMOSD**	**HC**	**P**	**NMOSD**	**HC**	**P**
FAZ-area (FA)	3.84 (1.74)	3.07 (1.06)	**0.050**	1.69 (0.49)	1.42 (0.52)	**0.020**	1.73 (0.55)	1.54 (0.59)	0.124
FAZ-circularity (FC)	0.70 (0.20)	0.71 (0.14)	0.685	0.94 (0.13)	0.99 (0.12)	0.079	0.92 (0.13)	0.92 (0.15)	0.954
FAZ-axial-ratio (FAR)	1.32 (0.80)	1.81 (0.63)	0.599	1.03 (0.20)	1.09 (0.13)	0.130	1.09 (0.18)	1.13 (0.14)	0.079
FAZ-roundness (FR)	44.16 (32.36)	36.06 (9.80)	0.106	41.69 (11.84)	37.67 (4.64)	0.340	38.46 (7.66)	36.15 (4.35)	0.025
FAZ-solidity (FS)	0.86 (0.06)	0.85 (0.06)	0.490	0.93 (0.03)	0.94 (0.04)	0.093	0.91 (0.04)	0.92 (0.05)	0.962
Direction-ratio	0.84(0.07)	0.83(0.06)	0.919	0.81 (0.04)	0.80 (0.04)	0.070	0.83 (0.07)	0.83 (0.05)	0.49
Direction-area	95386.17 (35612.57)	126975.41 (35612.57)	<**0.001**	194469.51 (65790.78)	253055.16 (60396.85)	<**0.001**	130898.89 (37292.13)	137133.29 (35682.46)	0.599
tortuosity	1.94 (0.36)	1.75 (0.28)	**0.015**	1.60 (0.20)	1.56 (0.18)	0.366	1.82 (0.29)	1.76 (0.30)	0.789
VAD	13.17(2.74)	15.50 (2.37)	<**0.001**	20.33 (5.18)	24.71 (5.16)	<**0.001**	15.97 (1.19)	16.58 (1.16)	0.462
VLD	4.71 (1.11)	5.71 (0.94)	<**0.001**	7.94 (2.04)	9.72 (1.92)	<**0.001**	5.97 (1.19)	6.22 (1.16)	0.497
FD	1.44 (0.05)	1.48 (0.04)	<**0.001**	1.55 (0.06)	1.59 (0.05)	**0.001**	1.49 (0.04)	1.50 (0.04)	0.557
B-num	114.76 (38.36)	142.18 (33.76)	**0.002**	191.81 (10.11)	240.26 (63.31)	**0.001**	138.07 (35.60)	146.06 (37.23)	0.762

The data with *P* value less than 0.05 as bold value, which generally means that the parameters they represent are significant.

To better explore the statistical correlations, the multivariate logistic regression analysis was performed on the NMOSD and HC data by adjusting their demographic factors including age and gender, as shown in Table 3. In both SVC and DVC layers, the OR < 1 indicates the FA measure in the NMOSD group increases when compared to the HC group, with the statistical significance of *P* < 0.050 for SVC and *P* < 0.01 for DVC. In contrast, decreases in terms of direction-area, VAD, VLD, FD, and B-num can be observed in the NMOSD group with OR>1 when compared to the HC group, with statistical significance of *P* < 0.01 on direction-area, VAD, VLD, FD, and B-num measures for both SVC and DVC, and *P* < 0.050 on tortuosity measure for SVC only. There is no noticeable difference in other metrics. Compared the results shown in Table 2 with Table 3, the main difference is that the decreasing trend of tortuosity in the NMOSD group of Table 2 switches to an increasing trend in Table 3, which suggests that the difference in tortuosity measurement between the two groups may be caused by confounding factors such as age and weight and has no direct relationship with NMOSD.

**Table 3:** Results of multivariate logistic regression analysis.

**Variable**	**SVC**			**DVC**			**IVC**		
	**OR**	**95%CI**	***P***	**OR**	**95%CI**	***P***	**OR**	**95%CI**	***P***
FAZ-area (FA)	0.633	0.427–0.937	**0.022**	0.233	0.082–0.659	**0.006**	0.454	0.191–1.082	0.075
FAZ-circularity (FC)	2.244	0.106–47.561	0.604	30.259	0.630–1453.895	0.084	1.46	0.048–44.625	0.828
FAZ-axial-ratio (FAR)	1.045	0.842–1.297	0.692	9.079	0.423–194.899	0.159	10.904	0.450–264.002	0.142
FAZ-roundness (FR)	0.98	0.954–1.006	0.126	0.939	0.875–1.009	0.085	0.923	0.847–1.006	0.067
FAZ-solidity (FS)	0.029	0.000–321.385	0.456	37190.185	0.036–3.837E+10	0.136	11.776	0.000–411645.646	0.644
Direction-ratio	0.092	0.000–277.978	0.092	6.01E-08	3.549E-14–0.102	**0.023**	0.036	5.817E-6–225.556	0.457
Direction-area	1	1.000–1.000	**0.001**	1	1.000–1.000	**0.001**	1	1.000–1.000	0.602
tortuosity	0.133	0.024–0.727	**0.02**	0.246	0.019–3.098	0.278	0.579	0.116–2.896	0.506
VAD	1.452	1.156–1.825	**0.001**	1.185	1.085–1.318	**0.002**	1.058	0.890–1.258	0.521
	2.59	1.474–4.552	**0.001**	1.616	1.217–2.145	**0.001**	1.143	0.749–1.745	0.535
FD	1.167E+09	1941.961–7.014E+14	**0.002**	15320922.78	248.051–9.463E+11	**0.003**	12.889	0.000–1107682.091	0.659
B-num	1.023	1.007–1.039	**0.005**	1.013	1.004–1.022	**0.003**	1.005	0.992–1.019	0.441

OR > 1: The increase in the coefficient has a reverse effect on the possibility of the sample belonging to NMOSD; OR = 1: The increase in the coefficient has no effect on the possibility of the sample belonging to NMOSD; OR <1:The increase in the coefficient has a positive effect on the possibility of the sample belonging to NMOSD. The data with *P* value less than 0.05 as bold value, which generally means that the parameters they represent are significant.

**Figure 3:**
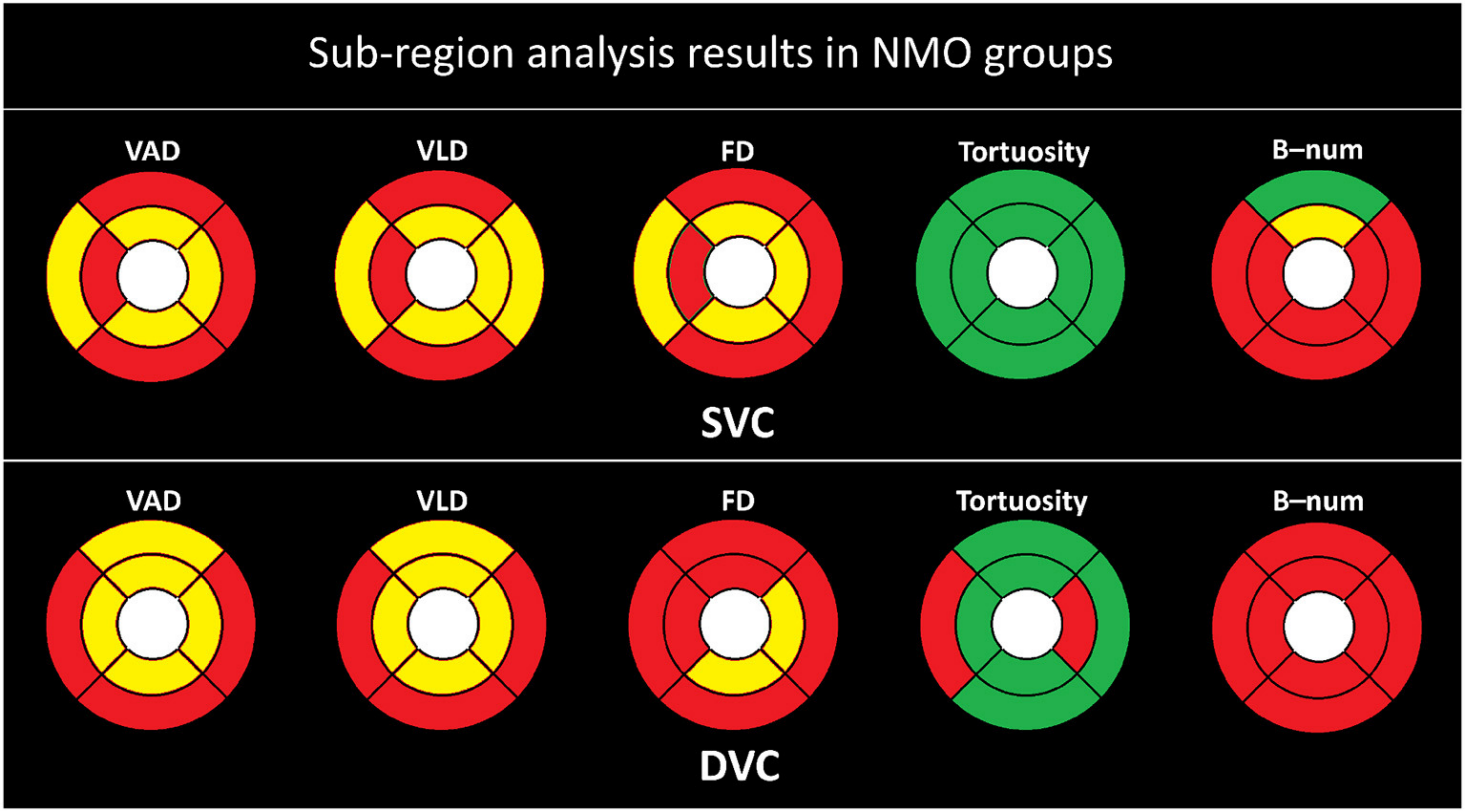
Sub-regional analysis of the NMOSD group. Here, the yellow color represents *P* ≤ 0.001, red represents *P* ≤ 0.050, and the green color represent *P* > 0.050.

To investigate the microvascular changes within different sub-regions, we performed the Student's *t*-test on different metrics between the NMOSD and HC groups, as shown in Figure 3. The yellow and red color, respectively, indicate the statistical significance of *P* < 0.001 and *P* < 0.050, while the green color represents there is no significant difference (*P*>0.050). From statistical results of the eight partitions of ETRDS, we can observe that there is more consistency on the measures of VAD, VLD, and FD between the SVC and DVC layers. All the partitions show statistical significance of *P* < 0.001 and *P* < 0.050 on these metrics. The B-num measure shows statistical significance of *P* < 0.050 on the whole DVC partitions, and it also presents significance of *P* < 0.001 and *P* < 0.050 on all the regions except the SE partition in the SVC. The tortuosity measure only presents significance on the TE and NI areas of the DVC with *P* < 0.050, while it obtains *P*>0.050 on all the other partitions of SVC and DVC.

### 3.2. Statistical comparisons between the non-ON and HC groups

To further study the effect of optic neuritis (ON) on the retinal microvascular changes of NMOSD patients. We divide the OCTA images of NMOSD patients into the ON and non-ON groups according to whether each of the eyes had optic neuritis. We finally select 25 ON eyes and 19 non-ON eyes to separately compared with the HC group. In Table 4, we can see the statistical results of all OCTA microvascular measures between the non-ON group and the HC group.

**Table 4:** Comparisons of OCTA metrics between HC and the non-ON eyes of NMOSD participants.

**Variable**	**SVC**			**DVC**			**IVC**		
	**Non-ON**	**HC**	***P***	**Non-ON**	**HC**	***P***	**Non-ON**	**HC**	***P***
FAZ-area (FA)	3.59 (1.31)	3.07 (1.06)	0.194	1.5 (0.55)	1.42 (0.52)	0.500	1.65 (0.55)	1.54 (0.59)	0.523
FAZ-circularity (FC)	0.69 (0.22)	0.71 (0.14)	0.817	0.94 (0.16)	0.99 (0.12)	0.261	0.91 (0.14)	0.92 (0.15)	0.79
FAZ-axial-ratio (FAR)	1.55 (1.10)	1.81 (4.63)	0.866	0.95 (0.18)	1.09 (0.13)	**0.002**	1.13 (0.17)	1.13 (0.14)	0.835
FAZ-roundness (FR)	32.32 (8.22)	36.06 (9.80)	0.287	45.76 (14.57)	37.67 (4.64)	**0.001**	36.75 (5.96)	36.15 (4.35)	0.679
FAZ-solidity (FS)	0.84 (0.07)	0.85 (0.06)	0.750	0.93 (0.04)	0.94 (0.04)	0.206	0.91 (0.04)	0.92 (0.05)	0.422
Direction-ratio	0.85 (0.08)	0.83 (0.06)	0.270	0.81 (0.04)	0.80 (0.04)	0.237	0.84 (0.05)	0.83 (0.05)	0.29
Direction-area	112396.77 (38514.02)	126975.41 (35612.57)	0.208	230685.61 (67930.71)	253055.16 (60396.85)	0.238	129787.94 (39670.54)	137133.29 (35682.46)	0.509
Tortuosity	1.89 (0.27)	1.75 (0.28)	0.193	1.58 (0.11)	1.56 (0.18)	0.782	1.85 (0.21)	1.76 (0.30)	0.293
VAD	14.41 (2.82)	15.50 (2.37)	0.221	23.08 (5.35)	24.71 (5.16)	0.305	15.96 (3.15)	16.58 (1.16)	0.484
VLD	5.23 (1.15)	5.71(0.94)	0.182	9.07 (2.07)	9.72 (1.92)	0.266	5.94 (1.25)	6.22 (1.16)	0.426
FD	1.47 (0.04)	1.48 (0.04)	0.308	1.58 (0.04)	1.59 (0.05)	0.520	1.49 (0.05)	1.50(0.04)	0.551
B-num	127.11 (38.81)	142.18 (33.76)	0.233	218.36 (50.47)	240.26 (63.31)	0.238	132.28 (29.76)	146.06 (37.23)	0.208

The data with *P* value less than 0.05 as bold value, which generally means that the parameters they represent are significant.

Specifically, there is no significant difference between the non-ON group and the HC group in the SVC layer of the OCTA data. In the DVC layer, the measures of FAR and FR in the non-ON group show significant differences from those of the HC group, with *P* < 0.01, while the other OCTA microvascular measures are not significantly different from each other, which indicates that the shape of FAZ in the DVC layer of the non-ON group may present pathological changes.

**Figure 4:**
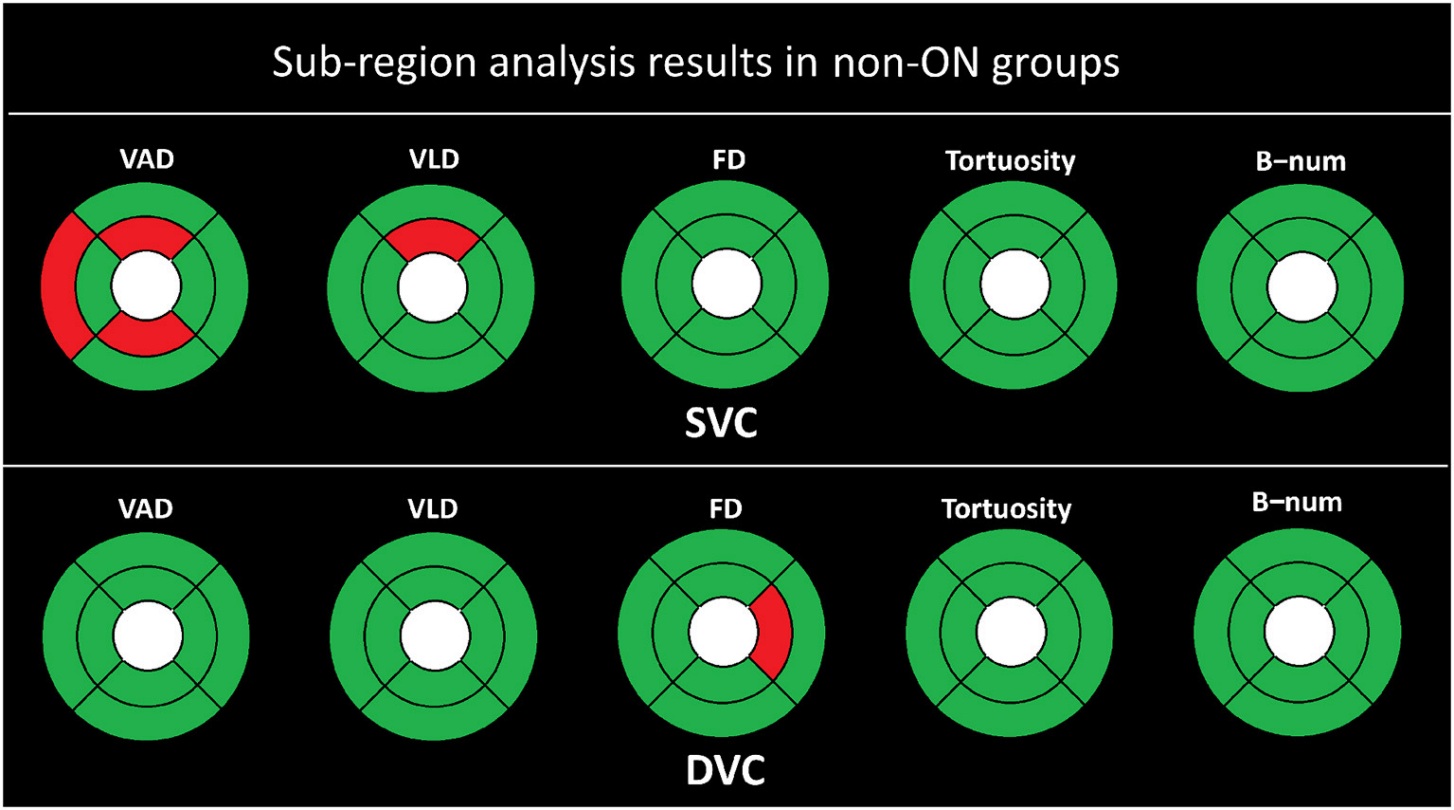
Sub-region analysis results of the non-ON groups. The red represents *P* ≤ 0.050, and the green color represents *P* > 0.050.

As shown in Figure 4, there is in general no strong association between the microvascular changes of the non-ON group and the HC group. The FD, tortuosity, and B-num in the SVC, and the VAD, VLD, tortuosity, and B-num in the DVC show no statistical difference between the two groups. For the measurements of VAD, only the TE, SI, and II partitions show a statistical significance of *P* < 0.050 in the SVC, while for the VLD, only the SI partition presents a significance of *P* < 0.050 in the SVC. For the FD, only the NI partition shows a statistical significance of *P* < 0.050 in the DVC.

### 3.3. Statistical comparisons between the ON and HC groups

The comparisons of OCTA microvascular metrics between the ON group and the HC group are provided in Table 5. Compared to the SVC layer of the HC group, the ON group shows remarkable increases in the measures of FA, FR, and tortuosity, with *P* < 0.050, while it presents significant decreases in the measures of direction-area, VAD, VLD, FD, and B-num, with *P* ≤ 0.001. The other metrics including FC, FAR, FS, and Direction-ratio remain unchanged. As for the DVC layer, only the FA measure in the ON group has a significant increase with *P* < 0.01, while the measures of direction-area, VAD, VLD, FD, and B-num show decreases, with *P* < 0.001. The other metrics including FC, FAR, FR, FS, direction-ratio, and tortuosity present no significant difference. Those findings imply that the retinal microvascular changes in OCTA images are strongly correlated with the progression of optic neuritis in the diseased eye. While it should be noted that some research groups believe that macular microvascular alterations arise independently of the occurrence of ON in NMOSD (Wei et al., 2021).

**Table 5:** Comparisons of OCTA metrics between HC and the ON eyes of NMOSD participants.

**Variable**	**SVC**			**DVC**			**IVC**		
	**ON**	**HC**	***P***	**ON**	**HC**	***P***	**ON**	**HC**	***P***
FAZ-area (FA)	3.97 (1.97)	3.07 (1.06)	**0.020**	1.82 (0.44)	1.42 (0.52)	**0.004**	1.79 (0.55)	1.54 (0.59)	0.124
FAZ-circularity (FC)	0.69 (0.19)	0.71 (0.14)	0.689	0.93 (0.11)	0.99 (0.12)	0.098	0.92 (0.13)	0.92 (0.15)	0.954
FAZ-axial-ratio (FAR)	1.19 (0.57)	1.81 (4.63)	0.595	1.09 (0.19)	1.09 (0.13)	0.925	1.06 (0.19)	1.13 (0.14)	0.079
FAZ-roundness (FR)	50.83 (38.83)	36.06 (9.80)	**0.015**	38.34 (8.00)	37.67 (4.64)	0.673	39.87 (8.74)	36.15 (4.35)	0.025
FAZ-solidity (FS)	0.87 (0.05)	0.85 (0.06)	0.232	0.93 (0.04)	0.94 (0.04)	0.175	0.92 (0.05)	0.92 (0.05)	0.962
Direction-ratio	0.82 (0.07)	0.83 (0.06)	0.504	0.81 (0.05)	0.80 (0.04)	0.092	0.82 (0.08)	0.83 (0.05)	0.49
Direction-area	85817.71 (31093.79)	126975.41 (35612.57)	<**0.001**	164644.48 (47683.01)	253055.16 (60396.85)	<**0.001**	131813.79 (36427.00)	137133.29 (35682.46)	0.599
Tortuosity	1.98 (0.41)	1.75 (0.28)	**0.018**	1.62 (0.26)	1.56 (0.18)	0.294	1.79 (0.35)	1.76 (0.30)	0.789
VAD	12.47 (2.52)	15.50 (2.37)	<**0.001**	18.07 (3.88)	24.71 (5.16)	<**0.001**	15.99 (2.88)	16.58 (1.16)	0.462
VLD	4.41 (1.00)	5.71 (0.94)	<**0.001**	7.01 (1.52)	9.72 (1.92)	<**0.001**	6.00 (1.18)	6.22 (1.16)	0.497
FD	1.42 (0.05)	1.48 (0.04)	<**0.001**	1.52 (0.05)	1.59 (0.05)	<**0.001**	1.49 (0.04)	1.50 (0.04)	0.557
B-num	107.81 (37.37)	142.18 (33.76)	**0.001**	169.94 (52.37)	240.26 (63.31)	<**0.001**	142.82 (40.04)	146.06 (37.23)	0.762

The data with *P* value less than 0.05 as bold value, which generally means that the parameters they represent are significant.

**Figure 5:**
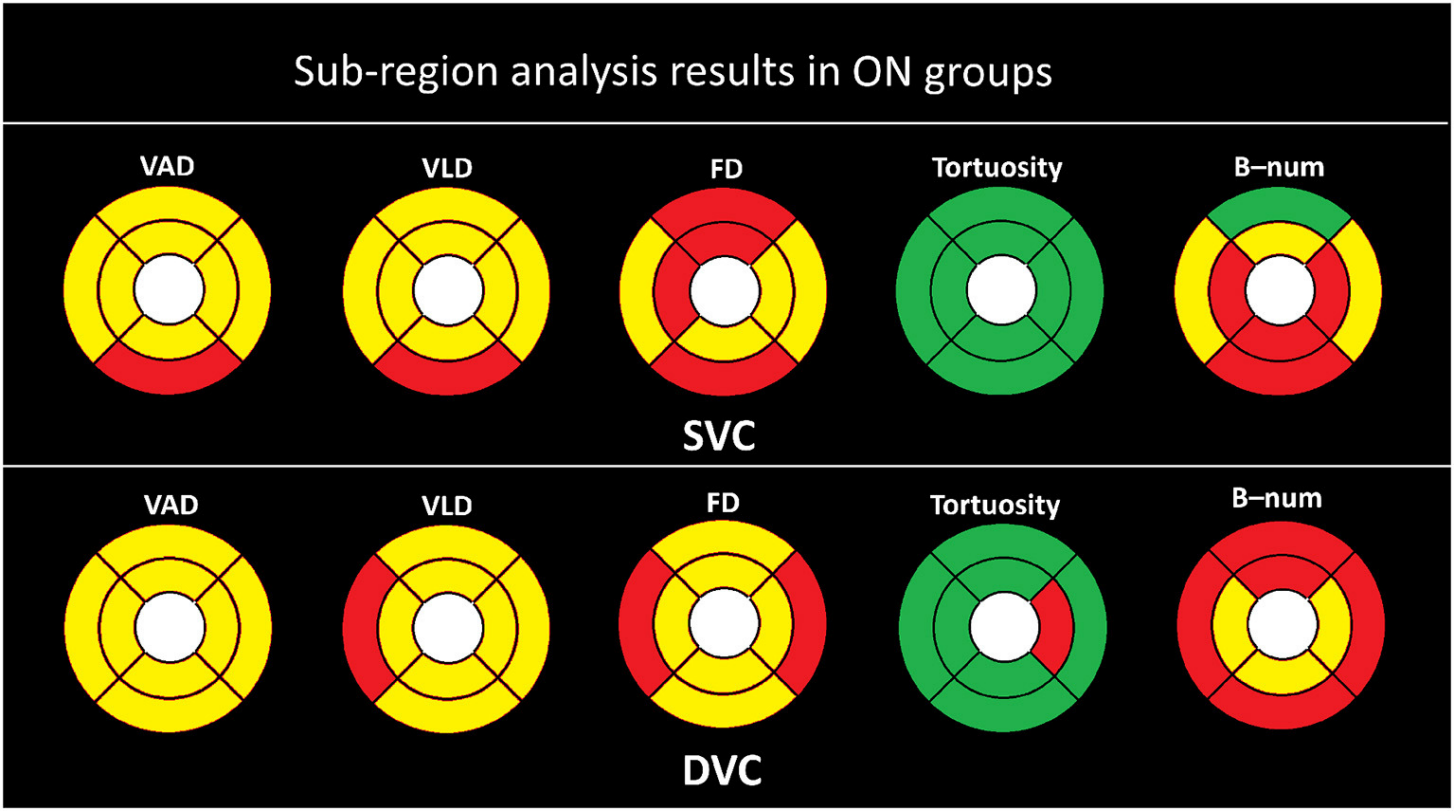
Sub-region analysis results in ON groups. Here, the yellow color represents *P* ≤ 0.001, red represents *P* ≤ 0.050, and the green color represent *P* > 0.050.

As shown in Figure 5, the statistical results are clearly more significant in the ON group than in the non-ON group when compared with the HC group. We can clearly observe that almost all partitions of the SVC and DVC present statistical significance of *P* < 0.001 and *P* < 0.050 in terms of the measures of VAD, VLD, FD, and B-num, while only the SE partition of the B-num metric in the SVC has no statistical difference between the ON and HC groups. For the tortuosity measure, only the NI partition in the DVC shows a statistical significance of *P* < 0.050 while the others had no difference.

## 4. Discussions

The tables above show that the foveal avascular zone (FAZ) areas of the neuromyelitis optica spectrum disorder (NMOSD) group are generally higher than those of the healthy control (HC) group. In most areas, the direction-area, vessel area density (VAD), vessel length density (VLD), fractal dimension (FD), and B-num of the NMOSD group are lower than those of the HC group. These results suggest that patients in the NMOSD group had an overall degeneration of the macular microvasculature, with decreases in both microvascular density and complexity. There is no significant difference in terms of tortuosity, flow complexity (FC), flow area ratio (FAR), flow rate (FR), flow symmetry (FS), and direction-ratio between the NMOSD group and the HC group. The sub-regional analysis in all figures also showed that there is no significant difference in the tortuosity of all partitions in SVC. The only signs that may indicate tortuosity changes are from the temporal superior and nasal inferior partitions in deep vascular complex, as shown in Figure 3. This indicates that the tortuosity measure is generally consistent in different areas and NMOSD may not be able to cause remarkable tortuosity variations.

Moreover, we divided the optical coherence tomography angiography (OCTA) images of neuromyelitis optica spectrum disorder (NMOSD) patients into the non-optic neuritis (ON) group and the ON group based on their history of optic neuritis. The comparisons with the healthy control (HC) group were respectively performed with respect to the two groups. From Table 4, we can observe that the differences in all OCTA microvascular metrics between the non-ON group and the HC group only exist in the flow area ratio (FAR) and flow rate (FR) of the deep vascular complex (DVC) layer. From Figure 4, we can see that there are possible signs of vessel area density (VAD) changes in the temporal superior (TE), superior inferior (SI), and inferior inferior (II) partitions, and vessel length density (VLD) changes in the SI. There is no obvious sign of microvascular changes in the other metrics and sub-regions. These findings may suggest that there is no damage to the microvasculature in the DVC layers, but changes could happen in the foveal avascular zone (FAZ) shape of the DVC in the non-ON group. Possible minor changes may appear in the superficial vascular complex partitions but not extensive. Besides that, the above results also indicate that even on the side with normal vision (i.e., non-ON side), the retina of NMOSD patients still undergoes some changes.

The FAZ shape changes found in the eyes of the normal side of NMOSD patients may reveal that NMOSD has no effect on the non-ON side, as previously generally recognized, but has a pathological response that cannot be easily detected. Because the eye on the unaffected side of NMOSD patients tends to gradually transform into an ON eye with repeated relapses, it is possible that changes found in the unaffected eye can predict the onset or relapse of NMOSD as an early feature, which depends on further research to confirm and may have important significance in clinical diagnosis and prevention.

As for the ON group of NMOSD patients, its OCTA microvascular characteristics are very similar to those of the NMOSD group, as shown in Figures 3, 5. The major changes are reflected in the larger FA and smaller microvascular density and complexity (i.e., VAD, VLD, and FD) compared with the HC group, as shown in Table 5. These findings indicate that the microvascular damage in NMOSD patients is mainly concentrated on the side of the onset of optic neuritis, which is consistent with the findings of others (Huang et al., 2018; Huang, 2020).

In summary, the OCTA images of NMOSD patients during the pre-onset period of optic neuritis mainly showed changes in the shape of FAZ in the deep layers of the retina, while the OCTA images of NMOSD patients after the onset of optic neuritis are mainly manifested as an increase in the FAZ area and a decrease in the microvascular density around the macula. According to other studies (Green and Cree, 2009; Hinson et al., 2012; Kaufhold et al., 2013), the appearance of the shape change of FAZ may indicate the existence of a certain degree of edema in the tissues near FAZ, and the pathological mechanism of damage to the microvasculature can generally cause the loss of vascular tissue. Interestingly, a series of studies have demonstrated that retinal tissue edema in NMOSD patients is highly correlated with AQP4 protein (Matsushita et al., 2009; Juenemann et al., 2015). In conclusion, such edema is uncommon in MS patients, suggesting that changes in the shape of the FAZ in the unaffected eye may serve as evidence to distinguish NMOSD from MS (Nakamura et al., 2009).

The biological conditions behind these pathological changes can be explained by several studies (Weinshenker et al., 2006; Ratelade et al., 2011; Jarius and Wildemann, 2013; von Glehn et al., 2014; Ashtari et al., 2021). In addition, researchers found retinal vessel loss might occur during NMOSD and might be linked to astrocyte damage and poor visual performance (Aly et al., 2022). The current research results show that the effect of NMOSD on the retina is mainly caused by the combination of NMO-BIG and AQP4 in astrocytes of the retinal nerve fiber layer (Brosnan and Raine, 2013; Vaknin-Dembinsky et al., 2014; Sofroniew, 2015; Chen et al., 2020). Such a combination can result in a series of cascade reactions, including changes in cellular water permeability, interactions with other types of glial cells, and abnormal immune reactions. The change of water permeability may cause edema, and an abnormal autoimmune response leads to apoptosis. Thus, it is possible that changes of water permeability in the glial cells cause edema around the FAZ, which is eventually presented as changes in the shape of the FAZ on the unaffected side. The immune reaction may lead to abnormal apoptosis in the retina, which results in a decrease of vessel density and an increase of the FAZ area in OCTA images.

However, the reason the widespread presence of NMO-IGg in the blood only causes one side of retinal damage is currently unknown. There are currently fewer studies on the different manifestations of NMOSD in diseased and non-affected eyes, and they focused more on OCT rather than OCTA (Feng and Hitam, 2020). In previous studies, no one mentioned the changes of retina on the non-ON side of NMOSD patients, while our study firstly found that there are changes in the shape of FAZ in the deep vessels of the NMOSD patients.

This study has several limitations. First, the number of participants was still relatively small as NMOSD is a rather rare disease. A cross-sectional study did not measure changes in retinal microvascular parameters over time or disease progression, and did not study disease disability levels, such as EDSS and NHISS, etc. Effects on retinal parameters. Longitudinal studies in larger cohorts need to be performed to determine whether these findings are completely reliable for identifying NMOSD patients in the pre-clinical stage. Second, data on eyeball axial length, usually used to identify myopia, was not acquired in this study: this may influence the area of OCTA captured, and thus introduce a bias in estimating the vascular parameters. However, the effect of the axial length should be very limited, as images captured from the elderly with highly pathological myopia are often of poor quality and therefore were excluded from our analysis. Third, our calculation of vessel tortuosity and bifurcation number is based on the projection of the three-dimensional vessel structure on two-dimensional, and we do not have distinguish different vessel structures. This has a certain negative impact on the accuracy of our calculations. In subsequent studies, we hope to segment retinal microvessels and FAZ directly based on 3D raw data, so as to calculate these parameters more accurately. Finally, several issues exist regarding the taxonomic comparison of ON versus non-ON retinas in this study. We only use whether optic neuritis has occurred as a basis for classification, without considering the number and severity of onset (?). Furthermore, we did not rule out that the morphological changes of the FAZ on the non-ON side may arise from subclinical effects on the ON side. These problems have raised certain challenges to the reliability of our conclusions, and the solution to these problems depends on further clinical research and innovations in analytical methods.

## 5. Conclusion

In this study, we established a complete framework for the processing and analysis of retinal microvascular biomarkers from optical coherence tomography angiography (OCTA) data. We provided accurate retinal vascular segmentation and foveal avascular zone (FAZ) segmentation techniques to extract key structures relevant to neuromyelitis optica spectrum disorder (NMOSD) disease. We applied efficient biomarker extraction methods to measure 12 important microvascular metrics from the segmentation results. Logistic regression analysis was performed to explore the most sensitive measures of high statistical significance between the NMOSD and healthy control (HC) groups. In addition, we specifically designed the sub-regional analysis routine to study the microvascular changes in a more localized manner. In general, we can summarize that the pathological changes mainly occur in the ON side, and the internal rings close to the FAZ present more significant microvascular variations than the external ring. These findings provide us with the possibility of integrating valuable retinal microvascular features for the early diagnosis of NMOSD.

## Data availability statement

The raw data supporting the conclusions of this article will be made available by the authors, without undue reservation.

## Ethics statement

The studies involving human participants were reviewed and approved by Cixi Institute of Biomedical Engineering, Chinese Academy of Sciences. The patients/participants provided their written informed consent to participate in this study.

## Author contributions

JG, DZ, and YZ contributed to conception and design of the study. DZ and JL organized the database. JG performed the statistical analysis. JG and JZ wrote the first draft of the manuscript. DZ and YG wrote sections of the manuscript. All authors contributed to manuscript revision, read, and approved the submitted version.
